# Laboratory Diagnosis of Antiphospholipid Syndrome in Anticoagulated Patients

**DOI:** 10.3390/biomedicines11061760

**Published:** 2023-06-19

**Authors:** Armando Tripodi, Erica Scalambrino, Marigrazia Clerici, Flora Peyvandi

**Affiliations:** 1 Fondazione IRCCS Ca’ Granda Ospedale Maggiore Policlinico, Angelo Bianchi Bonomi Hemophilia and Thrombosis Center, 20122 Milan, Italy; 2 Fondazione Luigi Villa, 20122 Milano, Italy; 3 Department of Pathophysiology and Transplantation, Università Degli Studi di Milano, 20122 Milano, Italy

**Keywords:** lupus anticoagulant, warfarin, direct oral anticoagulants, heparins, aPTT, dRVVT

## Abstract

The laboratory diagnosis of antiphospholipid syndrome (APS) requires the measurement of solid-phase antibodies to cardiolipin or β2-Glycoprotein-I and the search for lupus anticoagulant (LA). The diagnosis of patients whilst on anticoagulation is impaired by the difficult interpretation of results, at least for LA, owing to the fact that prolongations of clotting times induced by LA superimpose those induced by anticoagulants. This is a matter of concern as treating physicians very often need to know the APS status of their patients to make a decision on secondary antithrombotic prophylaxis. This article aims to review the effect brought about by anticoagulants on APS diagnosis and discuss the options that can be used to overcome such an effect.

## 1. Introduction

**Figure 1:**
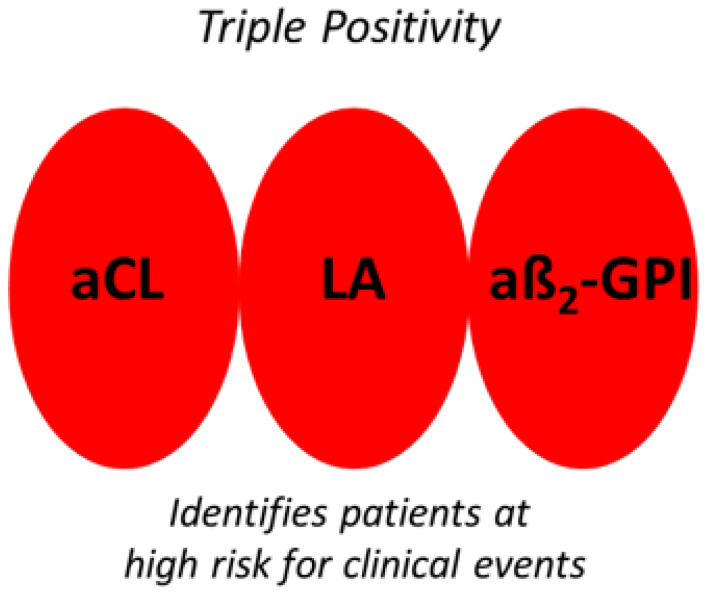
Schematic representation of triple-positive APS patients (i.e., concomitant presence of LA, low–medium titers of aCL and aβ2-GPI). The presence of persisting triple positivity poses a high risk for the development of clinical events. In contrast, isolated positivity of one or two components poses a lower risk. aCL, anticardiolipin. LA, lupus anticoagulant. aβ2-GPI, antiβ2-Glycoprotein-I.

Antiphospholipid syndrome (APS) is an autoimmune condition characterized by the presence of antibodies directed against negatively charged phospholipids in combination with proteins. The presence of these antibodies is very often associated with thrombosis (venous and/or arterial) and pregnancy complications. The laboratory diagnosis of APS is paramount as persistent positivity candidates patients for long-term anticoagulation. There is no single laboratory test to diagnose APS, and according to the current state of the art, laboratory diagnosis is performed by measuring the titer of antibodies to cardiolipin (aCL) and β2-Glycoprotein-I (aβ2-GPI), and by searching for the presence of lupus anticoagulant (LA) [1]. Positivity for one of the above is sufficient to qualify for the laboratory diagnosis of APS. Triple positivity (i.e., concomitant presence of aCL, aβ2-GPI and LA) identifies those patients at a greater risk of clinical events than either single or double positivity (Figure 1).

Until recently, the laboratory diagnosis of APS was deemed not strictly needed when patients were already started on anticoagulation because of acute venous thromboembolism. Indeed, this information was not deemed essential as the treatment of acute venous thromboembolism is similar regardless of the causative nature of the event. Acute venous thromboembolism is in fact treated with low-molecular-weight heparin (LMWH), followed by secondary prophylaxis with vitamin K antagonists or direct oral anticoagulants (DOAC). More recently, some of the DOAC have been licensed to be used for treatment of acute venous thromboembolism directly without initiation with LMWH. However, in recent years, two interventional clinical trials have been carried out whereby patients with triple positivity for APS (i.e., concomitant aCL, aβ2-GPI and LA) have been randomized to receive either vitamin K antagonists or rivaroxaban (an anti-factor Xa direct inhibitor). The aim of both studies was to ascertain if rivaroxaban was at least as effective and safe as the comparator (i.e., vitamin K antagonists) to prevent recurrent thrombosis and occurrence of bleeding. One of the two studies was prematurely interrupted, as in the rivaroxaban arm, there was an excess of events. Indeed, thromboembolic events were recorded in 12% of patients randomized to rivaroxaban as opposed to none in those randomized to vitamin K antagonists [2]. The other study was completed, and the results were similar [3]. Essentially, both studies showed that rivaroxaban is not effective to prevent recurrent thrombosis. Although the other DOAC have not yet been investigated, the European regulatory authority for medicines (i.e., EMA) issued a warning against the use not only of rivaroxaban, but of all the DOAC in patients with triple-positive APS. Accordingly, for these patients, the drugs of choice should be vitamin K antagonists until new data are available [4]. The above situation requires that patients with acute venous thromboembolism of unknown origin, who have been started on DOAC treatment must be referred as soon as possible to the laboratory for diagnosis of APS and, if triple positive, must be promptly switched to vitamin K antagonists to prevent recurrent events.

It should, however, be realized that in addition to DOAC, most anticoagulant drugs may have detrimental effects on APS laboratory diagnosis. This article aims to review the effect on APS laboratory diagnosis that should be expected based on each of the anticoagulant drugs that have been administered at the time of blood sampling. The results and conclusions of the overview are based on data from the literature and on personal experience.

## 2. Anticardiolipin (aCL) and Anti-β2-Glycorotein-I (aβ2-GPI)

The search for aCL and aβ2-GPI is performed via immuno-chemistry assays, whereby antibodies are detected by exposing plasma samples to the relevant antigen immobilized on solid-phase surfaces (i.e., plastic plates). The antibody–antigen complex is then detected with secondary specific antibodies conjugated with peroxidase by means of sandwich assays, called enzyme-linked immunosorbent assays (ELISA), or more recently through chemiluminescence assays [5]. ELISA and chemiluminescence assays possess advantages and disadvantages. ELISA, although sufficiently sensitive and specific, are poorly suitable for automation and are time consuming (at least 2 h to obtain results). Chemiluminescence assays are probably more sensitive and specific than ELISA, are highly automated, but require dedicated instruments, are less demanding than ELISA in terms of handling plasma samples and reagents, and the results are ready in a few minutes. Chemiluminescence assays are, however, more expensive than ELISA. There are many commercially available assays for aCL and aβ2-GPI that use different reagents and standards, and therefore, they are likely to give results that are poorly comparable when they come from different laboratories [6]. This is detrimental for the laboratory diagnosis and management of APS patients, as it is implicitly difficult to establish cut-off values for diagnosis and management that can be generalized to all patients and laboratories. However, a distinct advantage of solid-phase antibody detection is the fact that they (by definition) are not affected by any type of anticoagulation. Unfortunately, however, they cannot be used as standalone assays to define triple-positive patients without the detection of LA (see above).

## 3. Lupus Anticoagulant (LA) Detection

LA is a class of heterogenous antibodies that prolong in vitro such phospholipid-dependent coagulation tests as the activated partial thromboplastin time (aPTT)-derived tests and the dilute Russell viper venom test (dRVVT). Unfortunately, there are no specific tests to diagnose the heterogenous family of LA. Hence, laboratory diagnosis is based on indirect evidence. The guidelines issued by the Scientific and Standardization Committee of the International Society on Thrombosis and Haemostasis (ISTH) stipulate that LA detection should be performed by means of the aPTT or congener tests and dRVVT [7]. LA is considered positive when either aPTT, dRVVT or both meet three iterative diagnostic criteria (i.e., screening, mixing and confirmation). The screening criterion calls for one or both tests displaying clotting times above the cut-off value established in the laboratory. The mixing criterion calls for the persisting clotting time prolongation upon mixing (in the proportion 1:1) patient and normal plasma. Finally, confirmation calls for normalization of the clotting time prolongation upon repeating the screening test with increased phospholipids concentration in the assay system. 

**Figure 2:**
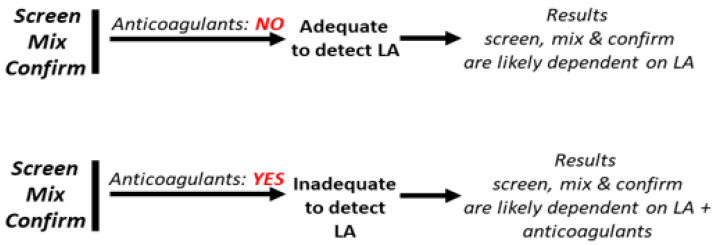
LA testing requires performance of three procedures. Screen: meant to provide evidence that one (or both) of the phospholipid-dependent tests (aPTT, dRVVT) have clotting times that are beyond the upper limits of the reference range. Mix: to provide evidence that the prolongations of the clotting time of the screen tests persist after mixing patients’ and normal plasma. Confirm: to provide evidence that the prolongations of the clotting times of the screen tests revert to normal while repeating the test upon increasing the concentrations of phospholipids. The procedures screen, mix and confirm are adequate to detect LA in the absence of anticoagulant drugs, as their results are likely dependent on LA only. Conversely, screen, mix and confirm are inadequate to detect LA in the presence of anticoagulant drugs, as their results are dependent on both LA and anticoagulant drugs. LA, lupus anticoagulant. aPTT, activated partial thromboplastin time. dRVVT, dilute Russel viper venom test.

However, it should be appreciated that the above diagnostic criteria do not work appropriately when patients have already been started on anticoagulants. In these conditions, the interpretation of screening, mixing and confirmation may in fact prove difficult because the clotting time prolongation due to LA superimposes that induced by the anticoagulant drugs (Figure 2). The following paragraphs aim to provide an overview of the interference that each of the main anticoagulant drugs used for the treatment of thrombosis may bring about in LA detection and the way to resolve or minimize their effects.

### 3.1. Low-Molecular-Weight Heparin (LMWH)

LMWH is a fast-acting antithrombotic drug, which works in combination with plasma antithrombin. It is often stated that LMWH does not prolong the aPTT and dRVVT. However, this is a misconception as depending on the ratio of anti-FIIa/anti-FXa displayed by different commercial brands, LMMW may occasionally prolong the aPTT and dRVVT. Ideally, each laboratory should be aware of or should assess the sensitivity of their own aPTT and dRVVT reagents for the effect of LMWH used for patients’ treatment. This can easily be performed by spiking pooled normal plasma with increasing amounts of the LMWH used for patients’ treatment and then measure the aPTT and dRVVT to ascertain the concentration of LMWH that is able to prolong the tests above the upper limits of the laboratory reference range. This information is useful to interpret the results of LA testing in patients who are on prophylaxis or treatment with LMWH. This said, LMWH is not a major problem for the laboratory diagnosis of LA, especially if blood is collected just before the next injection when the activity of LMWH is relatively low and, therefore, the effect on coagulation tests (if any) should be negligible.

### 3.2. Unfractionated Heparin (UFH)

UHF is known to prolong the aPTT and dRVVT, and hence, LA diagnosis cannot be reliably performed in patients who are treated with this drug, as the interpretation of the results would be inherently difficult, and in most instances, the occurrence of false positivity is very likely as shown by external quality assurance schemes [8]. However, it is anticipated that most of the commercial aPTT and dRVVT reagents, which are designed for LA detection, do contain optimal amounts of chemical substances (polybrene) or enzymes (heparinase) able to quench the activity of UFH up to 1.0 U/mL. The laboratory should be aware of the anti-heparin substances which are added to its own reagents.

### 3.3. Vitamin K Antagonists

Vitamin K antagonists are widely used to treat patients with cardiovascular diseases, and therefore, the chance for the laboratory to analyze samples from patients for LA whilst on these drugs is very likely. On these occasions, results should be interpreted with caution as the chance of obtaining false-positive or false-negative LA results is very likely. There are no simple strategies to overcome this problem, even though a few options have been proposed. They are discussed in the following paragraphs.

#### 3.3.1. Mixture of Patients and Pooled Normal Plasma 

This proposal calls for diluting the patient plasma into pooled normal plasma (proportion 1:1) and repeat LA testing on this mixture. The rationale for using such dilution rests on the concept that the coagulation factors in the pooled normal plasma are able to correct for the partial coagulation deficiency induced by vitamin K antagonists in the patient’s plasma. Although this procedure is widely adopted, it is not free from inconvenience. First, the correction of the abnormal coagulation time induced by vitamin K antagonists is variable and depends on the composition of the reagent (aPTT or dRVVT) used for LA detection. Another important issue is the quality of the pooled normal plasma that should be free of residual platelets and should contain individual coagulation factors with a potency close to 100 U/dL. Unfortunately, the chance of obtaining false-negative or false-positive results when adopting this option is very likely. Second, because of the dilution (1:1), the LA potency in the test plasma is reduced by 50%. Hence, weak LA could be lost at diagnosis. Third, the correction of prolonged clotting times induced by anticoagulant drugs is likely (with the above caveats) for vitamin K antagonists, but not for DOAC or heparins. This said, our personal recommendation is against using this procedure for LA detection. Whenever it is used, the results should be interpreted with caution.

#### 3.3.2. Taipan/Ecarin Snake Venoms-Based Methods

Venoms from certain snakes (*Oxyuranus scutellatus* or *Echis carinatus carinatus*), i.e., taipan and ecarin, respectively, possess distinct characteristics. Both can activate prothrombin (FII), but while the activity of taipan is dependent on phospholipids, ecarin is not. Hence, when used in combination in coagulation tests, the ratio of their clotting times is reportedly able to overcome the effect induced by anticoagulation. Until recently, there were scanty observations from the literature [9,10], and most were limited to patients on vitamin K antagonists. More recently, a relatively large multicenter study has been carried out to investigate the efficacy of the taipan/ecarin clotting time ratio to diagnose LA in plasma anticoagulated with vitamin K antagonists, DOAC or heparins. Most of the experiments were performed in normal pooled plasma spiked with increasing amounts of DOAC or heparins. Some were performed in standard plasma samples with known positivity for LA, and others were from anticoagulated patients with a historical knowledge of LA positivity. No samples from anticoagulated patients with documented positivity for LA were included. This cohort of patients is in fact inherently difficult to find and (most importantly) to characterize for true LA positivity, as there is no gold standard for LA detection during anticoagulation. With the above caveats, the study can be considered as a step forward to clarify the role of this assay in LA detection in patients on anticoagulants. It showed 78% sensitivity and 95% specificity for LA in patients with known APS [11]. One problem, which still waits to be resolved, is the availability of the assay in kit form and its standardization, both of which are badly needed to expand its use in clinical laboratories.

#### 3.3.3. Integrated LA Testing

Integrated tests call for testing plasma samples with dual aPTT and dRVVT tests: the first at low (screen) and the second at high (confirm) phospholipids concentrations. In the integrated tests, usually, the mixing procedure is skipped, and results are interpreted based on the ratio between screen and confirm. The higher the ratio, the greater the likelihood of LA positivity. The rationale of the procedure rests on the concept that in the presence of LA, the screening procedure displays prolonged clotting time, whereas the confirm procedure displays clotting time that reverts to normal. It is assumed that in the integrated procedures, the results of screen and confirm are affected to the same degree by anticoagulation, and therefore, their ratio is affected only by the presence of LA. An earlier investigation on plasma from patients (reportedly) positive for LA whilst on anticoagulation showed that integrated procedures employing aPTT or dRVVT were poorly affected by vitamin K antagonists or heparins [12], but there are no large independent studies confirming the efficacy of this strategy. Hence, results obtained when using this option should be interpreted with caution.

## 4. Direct Oral Anticoagulants (DOAC)

It is important to emphasize that the diagnosis of LA in patients who are anticoagulated with any of the DOAC presently used to treat cardiovascular diseases dramatically increases the rate of false positivity for LA and should therefore not be attempted [13]. On the other hand, mixing patients and normal plasma does not correct for the effect of anticoagulation. There are, however, options described in the recent literature that can be used to make a diagnosis in patients on DOAC, and these will be briefly discussed in the following paragraphs. Pros and cons of these options are summarized in Table 1.

### 4.1. DOAC Absorbents

**Figure 3:**
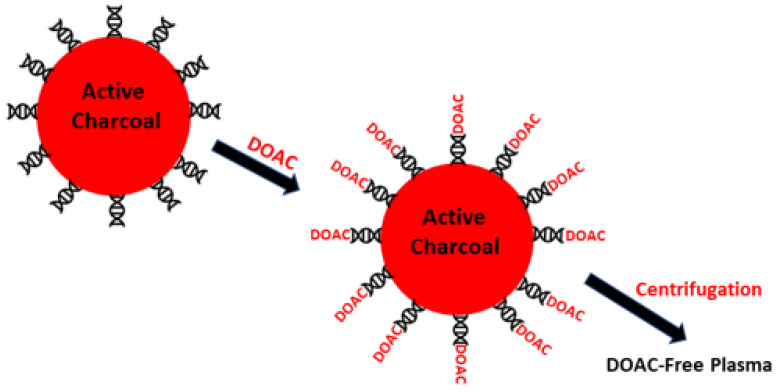
Schematic representation of DOAC absorbents. DOAC are absorbed on charcoal particles. After centrifugation, plasma is free from DOAC. The interference of DOAC on LA detection is minimized.

Chemical substances which are composed of active charcoal have been made available. These substances can help LA testing in patients on DOAC [14]. When mixed with patient plasma, they can absorb on their surface all the DOAC that are presently used for the treatment of cardiovascular diseases. After centrifugation, the supernatant plasma is free from DOAC, and coagulation factors as well as LA (if present) remain unaffected and can, therefore, be used to detect LA without interference (Figure 3). Presently, there are three commercially available products which can be used to remove DOAC: DOAC-Stop, DOAC-Remove and DOAC-Filter. A number of studies evaluated their efficacy to remove DOAC and to test for LA [15,16,17,18,19,20]. The first issue was easily evaluated, as the measurement of residual DOAC after charcoal exposure or filtration can be reasonably established by DOAC measurement before and after plasma exposure to the absorbents. However, the second, although investigated in many studies, suffers from the inherent difficulty (described above) of the availability of plasma samples from patients with documented LA whilst on anticoagulation. Cumulatively (with the above caveats), the absorbents showed acceptable capacity to remove DOAC from plasma. Whether DOAC absorbents leave the coagulation factors unaffected in the supernatant plasma after centrifugation is not completely established, as in some instances, the coagulation tests performed on LA-negative samples were somewhat affected by the exposure to the adsorbents. These results suggest that there may be unpredictable absorbance of coagulation factors or plasma substances other than DOAC [21]. Furthermore, as mentioned above, it is inherently difficult (if not impossible) to investigate plasma from patients with confirmed positivity for LA whilst on DOAC. Hence, a thorough and conclusive evaluation of the ability of DOAC removal and LA testing is still waiting. That said, we believe that DOAC absorbents are presently the most promising tools that the clinical laboratory can use to make a diagnosis of LA in patients on DOAC. It is important to emphasize that these absorbents are unable to remove heparins or to counteract the effect of vitamin K antagonists and that attempting LA detection without DOAC removal increases the risk of false-positive LA. In a recent study, we found that the rate of false-positive LA is about 88% in patients on rivaroxaban when tested with dRVVT [21].

### 4.2. DOAC Neutralizers

Recently, substances designed to inhibits DOAC have been developed and used for patients on DOAC at the time of bleeding because of suspected over dosage. These substances are idarucizumab and andexanet alfa, which are able to neutralize dabigatran and anti-factor Xa drugs, respectively. Both have been used as DOAC neutralizers in vitro before LA detection. Idarucizumab proved effective in neutralizing dabigatran in one study, and LA detection tests were apparently unaffected in the treated plasma [22,23]. Again, there is no evidence of its efficacy in plasma with confirmed LA positivity whilst on dabigatran. Another study investigated the applicability of andexanet alfa by means of a pooled normal plasma added with purified rivaroxaban. This plasma was supplemented with andexanet alfa and lyophilized in small aliquots. Aliquots of this plasma were sent to participants of a national external quality assessment scheme, who were asked to perform blind detection of LA by means of dRVVT. Most participants reported negative LA results for this plasma, but some others reported (false-) positive results. All in all, DOAC neutralizers, developed to treat patients on overdosage, proved variably effective for their use in LA testing. However, whatever their value, one should consider that they do not have a practical application in this context because of the relatively high cost.

## 5. Other Potential Procedures to Overcome Anticoagulation

A suitable procedure to overcome the influence of anticoagulation on LA testing is temporary discontinuation of anticoagulation for the time needed to clear all anticoagulants from circulation before LA testing. By definition, this would be by far the most effective procedure, but it is not free of inconvenience. The discontinuation of anticoagulation would be associated with unacceptable rates of recurrent thrombosis, especially in high-risk patients. This risk could be counteracted by replacing DOAC or vitamin K antagonists with LMWH, which presumably (see above) affects LA testing much less. However, clearing drugs from circulation, although prompt and effective for those drugs with short half-life such as DOAC or heparins, would require much more time for vitamin K antagonists. Finally, resuming anticoagulation after LA testing is practically demanding and not completely devoid of risk. All in all, the discontinuation of anticoagulation before LA testing is theoretically feasible, but it is not recommended by most experts, and the decision should be made for individual patients after careful consideration of the risk/benefit ratio. Another possible option could be to test plasma from patients on anticoagulation for the solid-phase antibodies aCL and aβ2-GPI, which are (by definition) not affected by anticoagulation, without caring too much for LA detection. If one or both tests are negative, then the patients can be considered as not being triple positive for APS and hence can be treated with DOAC. On the other hand, if both tests are positive, the patients might presumably be triple positive and therefore should be switched to vitamin K antagonists. 

## 6. Concluding Remarks

Laboratory operators and clinicians dealing with patients on treatment for cardiovascular diseases should be aware that anticoagulation of any type may be responsible for false-negative or false-positive LA results. Since LA detection is crucial to define triple positivity for APS, it is paramount that (whenever possible) blood samples for the laboratory diagnosis of APS be collected before starting anticoagulation. When this is not feasible, there are options that can be used for LA detection, but laboratory operators and clinicians should be aware that none of these options is 100% effective, and false-positive or false-negative results should occasionally be expected.

## Figures and Tables

**Table 1:** Summary of strategies for laboratory diagnosis of antiphospholipid syndrome in anticoagulated patients. aCL: anti-cardiolipin. aβ2-GPI: anti-β2 glycoprotein I. APS: antiphospholipid syndrome. VKA: vitamin K antagonists. DOAC: direct oral anticoagulants. UFH: unfractionated heparin. LMWH: low-molecular-weight heparin.

Strategy	Pros	Cons
aCL, aβ2-GPI	Not affected by anticoagulation	If used alone, they are not effective to diagnose triple-positive APS.
Mixture of patients and pooled normal plasma	Relatively simple; applicable only for patients on VKA	Possible false-positive or false-negative results.
Taipan/Ecarin snake venoms-based methods	Relatively simple	No conclusive evidence for efficacy. Difficult availability of standardized reagents.
Integrated LA testing	Relatively simple	No conclusive evidence on diagnostic efficacy stemming from large studies.
DOAC absorbents	Relatively simple	They remove DOAC, but there is no definitive evidence that they do not modify coagulation factors. Not effective for UFH or LMWH.
Neutralizers	Polybrene or heparinase quench UFH up to 1 U/mL	-
Other DOAC neutralizers	Idarucizumab or andexanet alfa neutralize in vitro dabigatran or anti-FXa drugs	No definitive evidence for their diagnostic efficacy. Very expensive.
Testing before starting anticoagulation	Relatively safe diagnostic procedure	Possible interference should be expected if testing is performed during acute thrombosis.
Discontinuation of anticoagulation	Relatively safe diagnostic procedure when anticoagulants are replaced by LMWH	The laboratory should be aware of the possible interference brought about by the brand of LMWH. Difficult in practice as it requires resuming anticoagulation after APS diagnosis.
